# A proposal to recognize investment in breastfeeding as a carbon offset

**DOI:** 10.2471/BLT.23.290210

**Published:** 2024-03-07

**Authors:** Julie Patricia Smith, Phillip Baker, Roger Mathisen, Aoife Long, Nigel Rollins, Marilyn Waring

**Affiliations:** a National Centre for Epidemiology and Population Health, Building #63, cnr Mills and Eggleston Rd, Australian National University, Canberra 0200, Australia.; b Sydney School of Public Health, University of Sydney, Sydney, Australia.; c Alive & Thrive, FHI 360, Hanoi, Viet Nam.; d Munster Technological University, Munster, Ireland.; e Department of Maternal, Newborn, Child and Adolescent Health and Ageing, World Health Organization, Geneva, Switzerland.; f Institute of Public Policy, Auckland University of Technology, Auckland, New Zealand.

## Abstract

Policy-makers need to rethink the connections between the economy and health. The World Health Organization Council on the Economics of Health for All has called for human and planetary health and well-being to be moved to the core of decision-making to build economies for health. Doing so involves valuing and measuring what matters, more and better health financing, innovation for the common good and rebuilding public sector capacity. We build on this thinking to argue that breastfeeding should be recognized in food and well-being statistics, while investments in breastfeeding should be considered a carbon offset in global financing arrangements for sustainable food, health and economic systems. Breastfeeding women nourish half the world’s infants and young children with immense quantities of a highly valuable milk. This care work is not counted in gross domestic product or national food balance sheets, and yet ever-increasing commercial milk formula sales are counted. Achieving global nutrition targets for breastfeeding would realize far greater reductions in greenhouse gas emissions than decarbonizing commercial milk formula manufacturing. New metrics and financing mechanisms are needed to achieve the health, sustainability and equity gains from more optimal infant and young child feeding. Properly valuing crucial care and environmental resources in global and national measurement systems would redirect international financial resources away from expanding carbon-emitting activities, and towards what really matters, that is, health for all. Doing so should start with considering breastfeeding as the highest quality, local, sustainable first-food system for generations to come.

## Résumé

Les responsables politiques doivent repenser les liens entre économie et santé. Le Conseil de l'Organisation mondiale de la Santé sur l'économie de la santé pour tous a demandé que le bien-être et la santé, aussi bien de l'humain que de la planète, soient désormais au cœur du processus de prise de décisions afin de créer des économies au service de la santé. Il est donc impératif d'identifier et de valoriser ce qui compte, d'accroître et d'optimiser le financement de la santé, d'innover pour le bien commun et de renforcer les capacités du secteur public. En partant de cette réflexion, nous plaidons pour une reconnaissance de l'allaitement dans les statistiques relatives à l'alimentation et au bien-être, et estimons que tout investissement réalisé dans ce domaine devrait être considéré comme un crédit-carbone dans le cadre des modalités financières mondiales liées aux systèmes économiques et sanitaires ainsi qu'à une alimentation durable. Les femmes allaitantes nourrissent la moitié des enfants en bas âge dans le monde avec d'immenses quantités de lait extrêmement précieux. Ces activités de soins ne sont pas comptabilisées dans le produit intérieur brut ou les bilans alimentaires nationaux, contrairement aux ventes de lait maternisé en constante progression. Atteindre les cibles mondiales de nutrition pour l'allaitement contribuerait davantage à réduire les émissions de gaz à effet de serre que décarboner la production de lait maternisé. De nouveaux paramètres et mécanismes de financement sont nécessaires pour bénéficier des avantages en matière de santé, de durabilité et d'équité qui découlent d'une meilleure alimentation des nourrissons et jeunes enfants. Valoriser correctement les principales ressources consacrées à l'environnement et aux soins dans les systèmes de mesure nationaux et mondiaux permettrait de détourner les moyens financiers internationaux du développement d'activités à fort taux d'émissions pour les rediriger vers ce qui compte vraiment, c'est-à-dire la santé pour tous. Et pour y parvenir, la première étape consisterait à reconnaître l'allaitement comme l'aliment de base, local, durable et de qualité pour les générations futures.

## Resumen

Los responsables de formular las políticas deben volver a plantearse las conexiones entre la economía y la salud. El Consejo sobre la Economía de la Salud para Todos de la Organización Mundial de la Salud ha pedido que la salud y el bienestar humanos y del planeta se sitúen en el centro de la toma de decisiones a fin de desarrollar economías para la salud. Esto requiere valorar y medir lo que importa, más y mejor financiación sanitaria, innovación para el bien común y reconstrucción de la capacidad del sector público. Nos basamos en este pensamiento para argumentar que la lactancia materna debería reconocerse en las estadísticas de alimentación y bienestar, mientras que las inversiones en lactancia materna deberían considerarse como una compensación de emisiones de carbono en los acuerdos globales de financiación para sistemas alimentarios, sanitarios y económicos sostenibles. Las mujeres lactantes alimentan a la mitad de los bebés y niños pequeños del mundo con inmensas cantidades de una leche muy valiosa. Este trabajo de cuidados no se contabiliza en el producto interior bruto ni en los balances alimentarios nacionales y, sin embargo, sí se contabilizan las ventas cada vez mayores de leche de fórmula comercial. Alcanzar los objetivos mundiales de nutrición para la lactancia materna supondría una reducción mucho mayor de las emisiones de gases de efecto invernadero que descarbonizar la fabricación de leche de fórmula comercial. Se necesitan nuevas métricas y mecanismos de financiación para lograr los beneficios en materia de salud, sostenibilidad y equidad de una alimentación más óptima de los bebés y los niños pequeños. Una valoración adecuada de los cuidados esenciales y de los recursos medioambientales en los sistemas de medición globales y nacionales redirigiría los recursos financieros internacionales lejos de la expansión de las actividades que emiten carbono, y hacia lo que realmente importa, es decir, la salud para todos. En este contexto, habría que empezar por considerar la lactancia materna como el sistema de primera alimentación de mayor calidad, local y sostenible para las generaciones futuras.

## ملخص

يحتاج واضعو السياسات إلى إعادة التفكير بشأن الروابط بين الاقتصاد والصحة. دعا مجلس اقتصاديات الصحة للجميع (Economics Health for All has) التابع لمنظمة الصحة العالمية، إلى وضع صحة ورفاهية الإنسان والكوكب في قلب عملية صنع القرار المتعلق ببناء اقتصاديات الصحة. ويتطلب القيام بذلك تقييم وقياس ما له تأثير على الأمر، وزيادة وتحسين التمويل الصحي، والابتكار من أجل الصالح العام، وإعادة بناء قدرات القطاع العام. ونحن نعوّل على هذا التفكير لدعم مبدأ إنه ينبغي وضع الرضاعة الطبيعية في الاعتبار فيما يتعلق بإحصاءات الغذاء والرفاهية، في حين ينبغي اعتبار الاستثمارات في الرضاعة الطبيعية بمثابة تعويض للكربون في ترتيبات التمويل العالمية للأنظمة الغذائية والصحية والاقتصادية المستدامة. تقوم السيدات المرضعات بإرضاع نصف عدد الرضع والأطفال الصغار في العالم، بكميات هائلة من الحليب عالي القيمة. لا يتم احتساب أعمال الرعاية هذه في الناتج المحلي الإجمالي، أو الميزانيات الوطنية للأغذية، بينما يتم احتساب مبيعات تركيبات الحليب التجارية، والمتزايدة باستمرار. إن تحقيق أهداف التغذية العالمية للرضاعة الطبيعية من شأنه أن يحقق تخفيضات أكبر بكثير في انبعاثات غازات الاحتباس الحراري، مقارنة بعملية نزع الكربون في تصنيع تركيبات الحليب التجارية. هناك حاجة إلى مقاييس وآليات تمويل جديدة لتحقيق مكاسب فيما يتعلق بالصحة والاستدامة والمساواة، من خلال التغذية المثالية للرضع والأطفال الصغار. إن التقييم السليم لموارد الرعاية الحيوية والموارد البيئية في أنظمة القياس العالمية والوطنية من شأنه أن يعيد توجيه موارد التمويل الدولية بعيداً عن الأنشطة المتوسعة المسببة لانبعاثات الكربون، ونحو ما له تأثير على الأمر بالفعل، وهو الصحة للجميع. إن القيام بذلك ينبغي أن يبدأ باعتبار الرضاعة الطبيعية كنظام الغذاء الأول المحلي الأعلى جودة، والمستدام للأجيال القادمة.

## 摘要

政策制定者需要重新思考经济与健康之间的联系。世界卫生组织全民健康经济理事会呼吁将人类和地球的健康和福祉作为决策核心,以建立促进健康的经济。要做到这一点,就需要评估和衡量重要的事项,增加健康筹资和改善筹资情况,为共同利益而进行创新,以及重建公共部门的能力。基于这一思路,我们认为母乳喂养应被列入食品和福祉统计数据的范畴,而在可持续食品、健康和经济系统的全球筹资安排中应被视为一种碳抵消行为。进行母乳喂养的妇女提供了大量宝贵的乳汁,滋养了世界上一半的婴幼儿。这种护理性工作未被计入国内生产总值或国家食品供求平衡表,但不断增长的商业配方奶粉销售额却被计入其中。与让商业配方奶粉生产做到脱碳相比,实现母乳喂养的全球营养目标将能更大程度地减少温室气体排放。需要新的衡量指标和筹资机制,以实现更优化的婴幼儿喂养,从而获得健康、可持续和公平的收益。在全球和国家衡量系统中正确评估关键的护理和环境资源,将使国际财政资源的分配从不断扩大的碳排放活动转向真正重要的事项,即全民健康。若要做到这一点,应先将母乳喂养视为子孙后代的最优质和可持续的当地第一食物系统。

## Резюме

Ответственным лицам необходимо переосмыслить связь между экономикой и здравоохранением. Совет Всемирной организации здравоохранения по экономическим аспектам «Здоровье для всех» призвал сделать здоровье и благополучие человека и планеты основой процесса принятия решений для построения экономики в интересах здоровья. Для этого необходимо оценить и измерить важность того, что имеет значение, увеличить и улучшить финансирование здравоохранения, внедрить инновации для общего блага и восстановить потенциал государственного сектора. Основываясь на этих соображениях, авторы статьи утверждают, что грудное вскармливание должно учитываться в статистике продовольствия и благосостояния. Инвестиции в грудное вскармливание должны рассматриваться как компенсация за выбросы углекислого газа в рамках глобальных механизмов финансирования устойчивых продовольственных, медицинских и экономических систем. Кормящие женщины обеспечивают половину детей грудного и раннего возраста в мире огромным количеством драгоценного молока. Этот труд по уходу не учитывается ни в валовом внутреннем продукте, ни в национальных продовольственных балансах, однако при этом учитывается постоянно растущий объем продаж молочных смесей. Достижение глобальных целей в области питания, связанных с грудным вскармливанием, приведет к гораздо большему сокращению выбросов парниковых газов, чем декарбонизация производства молочных смесей. Необходимы новые метрики и механизмы финансирования для достижения преимуществ в области здравоохранения, устойчивости и справедливости за счет более оптимального питания детей грудного и раннего возраста. Правильная оценка важнейших ресурсов по уходу и охране окружающей среды в глобальных и национальных системах измерения позволит перенаправить международные финансовые ресурсы не на расширение деятельности по выбросу углекислого газа, а на действительно важные аспекты – на здоровье для всех. Для этого следует начать с рассмотрения грудного вскармливания как наиболее качественной местной устойчивой системы первого питания для будущих поколений.

## Introduction

Amid widespread health system weaknesses and inequities exposed by the coronavirus disease 2019 (COVID-19) pandemic, the World Health Organization (WHO) Director-General tasked the WHO Council on the Economics of Health for All to lead a rethink of connections between the economy and health.

We build on the thinking of the council to propose a reorientation of cross-sectoral public policies on health and climate financing, based on countries’ fundamental human rights obligations to women and children on breastfeeding.

Breastfeeding makes vital contributions to realizing the right to the highest attainable standard of nutrition and health, the right to food and the right to life.^1^^,^^2^ Breastfeeding underpins child nutrition, health and survival, and women’s health;^3^ it is also a contributing factor for child health equity,^4^ while lack of breastfeeding adversely affects children’s cognitive development and lifetime opportunities.^5^ The immunological role of breastfeeding is so evident that it is considered the first vaccine by the United Nations Children's Fund (UNICEF) and WHO, both of which have called for wide-ranging public investments to enable breastfeeding.^6^ Yet during the COVID-19 pandemic, health service providers often disregarded protocols supporting breastfeeding.^7^ More infants were at risk of dying during the COVID-19 pandemic from lack of breastfeeding than from the disease.^8^ Ironically, new opportunities for commercial milk formula marketing were leveraged by industry as public capacities came under strain.^9^

Commercial milk formula was developed as a legitimate product to meet the nutrition requirements of infants who could not be breastfed.^10^ Yet today, more than half the world’s children receive substances other than breastmilk in their first six months of life.^11^ Commercial milk formula sales rise strongly with per capita gross domestic product (GDP) and expansion of the market economy.^12^ The 2023 *Lancet* series on breastfeeding documented the powerful role of modern marketing in this expansion.^13^ This role includes the distortion of science by the food industry to promote commercial milk formula to the public and in health-care systems. Global trade rules and regulatory regimes for intellectual property and investor protection are often exploited to discourage countries from implementing regulations constraining formula marketing.^9^^,^^14^^–^^16^ Limited public investment in health-care services and maternity protections that would otherwise enable breastfeeding creates opportunities for marketers to exploit the vulnerabilities of women and families.^13^

We suggest that breastfeeding illustrates how current thinking and systems undervalue what matters, inequitably distort investment priorities, and strengthen commercial drivers of health at the expense of true innovation, public health capacity and human and planetary health. Breastfeeding women^1^ nourish half the world’s infants and young children,^17^ but this fact is rarely captured in key economic metrics or resourced in national budgets. Yet expanding markets in high greenhouse gas-emitting dairy products is recognized and rewarded as commercial milk formula counts towards GDP growth, while the low-carbon footprint and immense productivity of breastfeeding women in producing vast quantities of highly valuable breastmilk is unrecognized and under-resourced.

Attempts to reform the current GDP growth-based paradigm to encompass the co-dependence of the natural world and health include incorporating environmental accounts into measures of the economy. The reform also includes creating carbon offset schemes to redirect financing and investment away from environmentally harmful economic activities and towards activities that reduce, remove or capture emissions.

In this paper, we build on these efforts to propose that breastfeeding investments be recognized as a carbon offset, to simultaneously help draw down the excessive production and consumption of commercial milk formula. This novel approach to valuing what matters and connecting the economy to health can be informed by new metrics for capturing the economic value that breastfeeding women generate. First, we illustrate the conceptual shortcomings of current economic accounting norms and conceptual frameworks by reference to breastfeeding, and outline new metrics for incorporating economic and environmental values of health for all into policy and practice. Second, we show how having new metrics for measuring women’s production of breastmilk can support new public investments in breastfeeding as a carbon offset, with significant gains and co-benefits for women’s, children’s and planetary health.

## Towards new metrics and indicators 

The concept of value underpinning the council’s final report is valuing planetary health, such as clean water, clean air and a stable climate; valuing the diverse social foundations and activities that promote equity; and valuing human health and well-being.^18^

GDP, the core economic metric of the United Nations (UN) System of National Accounts, was never intended even as a measure of economic progress and now powerfully contradicts these values. Statisticians, economists, feminists and environmentalists have extensively critiqued GDP over many years for its narrow definition of economic activity and material well-being, and for not measuring the degradation and depletion of the natural world. As the council points out, GDP includes goods and services that damage health and reduce social welfare.^18^ Feminist economists have criticized the failure of GDP to count women’s unpaid care and reproductive work as economically productive.^19^^,^^20^ Empirical studies show that this bias has misled policy-makers about underlying trends in economic performance and distributional equity.^21^^,^^22^

Studies in multiple and diverse locations have confirmed the important scale and monetary value of breastfeeding.^23^^–^^27^ Norway has included breastmilk in its national food statistics since 1994.^28^ Conceptual guidance provided in the *System of National Accounts 1993*^29^ provided the opportunity to include breastmilk in GDP as an own-account good (produced by households for their own use), yet milk produced by breastfeeding women is not yet counted in GDP in any country. This omission is because women’s breastfeeding and provision of breastmilk is rarely monetized, but commercial milk formula and commercial human milk products count in GDP as economically valuable because they are bought and sold.^27^ As disease and illness are more prevalent among women who cease breastfeeding prematurely and among children who are not sufficiently breastfed,^3^ higher commercial milk formula sales result in higher health-care costs and additional unpaid work burdens on caregivers.^30^^,^^31^ However, these additional health-care expenditures are measured by GDP as expanding the economy. Breastfeeding does not count in the System of National Accounts framework for human capital formation as women provide its economic benefits, including of higher intelligence, academic achievement and future productivity and earnings, free of charge.^32^ Similarly, GDP measurement continues to ignore the considerable and multiple environmental harms of commercial milk formula, including not only greenhouse gas emissions and water used during the product life cycle, but also various kinds of waste, pollution and contamination.^33^

New nutrition tools show this systemic bias in System of National Accounts metrics can be quantified by costing the hidden externalities of commercial milk formula. The Mothers Milk Tool^17^ estimates the quantities of breastmilk production and its monetary value using UNICEF data on infant and young child feeding practices, and a proxy value of 100 United States dollars (US$) per litre based on the price at which unprocessed breastmilk is exchanged by Norway’s milk bank network. The Cost of Not Breastfeeding Tool^30^ quantifies the health system costs and lost economic opportunities (that is, the hidden cost externalities of commercial milk formula) when breastfeeding and breastmilk is displaced in infant and young child diets. The Green Feeding Tool^34^ quantifies the substantial greenhouse gas emissions and water use impacts of commercial milk formula during its production and use for most low- and middle-income countries, using available data on infant and young child feeding practices for infants 0–6 months, and has functionalities including to calculate the carbon offset of policies and programmes that are known to increase breastfeeding rates.^34^

The international agencies responsible for the System of National Accounts have made attempts to change GDP formally in the UN system. In 1993, a revised System of National Accounts allowed inclusion of some non-market goods production and satellite accounts of non-market household services. This inclusion made it relatively straightforward to acknowledge breastmilk as a separate category in the System of National Accounts’ central product classifications, and for countries to acknowledge breastmilk in GDP by compiling households’ own-account production of goods.^27^ After the 2007 global financial crisis exposed the limits of GDP as an indicator of economic performance and social progress, the Stiglitz–Sen–Fitoussi Commission used breastmilk to illustrate the GDP-biased policy-making by excluding non-market production.^35^ The Commission’s work stimulated the Beyond GDP agenda to develop other indicators of well-being,^36^ and since 2009 UN agencies have included new guides for valuing unpaid household services and harmonizing national time use surveys.^37^ The System of Environmental-Economic Accounting^38^ offers international standards for integrating environmental and economic statistics with experimental ecosystem accounting for greenhouse gas emissions. Yet no country measures human milk production in GDP, applies time-use accounting for breastfeeding, or measures the health and environmental costs of commercial milk formula.

While the Organisation for Economic Co-operation and Development (OECD), in its Well-Being Framework,^39^ advocates for going beyond GDP, the measurement system and the market-focused paradigm behind it remains largely unchanged. The WHO Council on the Economics of Health for All concluded that modifications on GDP as the measure of progress cannot address the fundamental schism between the goal of health for all and what society values. Recently, the 2023 *Lancet* Series on Breastfeeding called for the adoption of an economic paradigm that views expenditure on breastfeeding protection, promotion and support as an investment with positive social, economic and environmental returns, and not as a cost.^13^ The series also called for better metrics to help address the care policy and resourcing deficits, and excessive work burdens for women that currently undermine breastfeeding.^13^

Time-use data are proposed by the council as an alternative metric so that resource distribution can reflect who does the most work.^40^ Breastfeeding and infant care is time intensive,^41^ and these productive activities can be captured through well-designed time-use surveys. Breastfeeding should be added to the OECD’s framework for both women and children, and measured in time-use statistics so women’s substantial investments in breastfeeding are better recognized.^39^

Governments could adopt policies recognizing women’s contribution to food production more broadly, to generate impetus for including breastmilk in food balance sheets, such as those as compiled by the Food and Agriculture Organization (FAO) of the United Nations internationally. This transformative change would fully align with the recent call by FAO for true cost accounting to internalize the health and environmental cost externalities generated by agrifood systems in general, and by commercial milk formula in particular, as discussed below.^36^

## Breastfeeding as carbon offsets

Strong scientific evidence exists of links between how infants and young children are fed and environmental harms. One kg of commercial milk formula generates around 11–14 kg of greenhouse gas, and uses more than 5 000 L of water during the product life cycle^42^, as well as multiple other harms to planetary health, such as land use change and biodiversity loss, antimicrobial resistance, zoonoses, air pollution and soil degradation.^33^^,^^43^ Achieving the global nutrition targets for breastfeeding would lead to far greater reductions in greenhouse gas emissions than decarbonizing commercial milk formula manufacturing.^44^ As well as mitigating climate change, breastfeeding also supports adaptation and builds resilience to disasters.^45^ Yet, country policies permit or even encourage expansion of the powerful commercial milk formula industry;^13^ current metrics are embedded in a GDP growth paradigm that valorizes expansion of markets, so commercial milk formula sales are more visible, valued and invested in. Reversing this thinking and realigning global financing and investment towards mitigation of harms to planetary and human health is needed.

Less than 3% of multilateral climate financing directly goes towards child-responsive activities.^46^ Furthermore, additional government investment of US$ 5.7 billion is needed to meet the global nutrition target for exclusive breastfeeding by 2025, but donor disbursements were only US$ 59 million in 2021.^47^

We propose a pathway for financing just and equitable policy change that conceives of breastfeeding as a carbon offset and reorients public investments towards this low-zero carbon activity.

Carbon or greenhouse gas accounting calculates and analyses how much carbon dioxide an individual, organization or country emits, and informs methods underpinning carbon markets.^48^ Underlying carbon accounting schemes is the concept of carbon pricing.^49^ Carbon pricing policies facilitate development of carbon offset schemes, wherein greenhouse gas emitters pay for the cost of offsetting their emissions by buying carbon credits to compensate for their greenhouse gas emissions. An example is the United Nations Carbon Offset Platform, which enables the purchase of offsets called Certified Emission Reductions to fund projects in low- and middle-income countries. 

Methods and platforms are available for carbon accounting and carbon offsetting at personal, business, national and international levels. Some carbon offset markets and programmes, such as the European Union Emission Trading Scheme, are legally mandated and compel companies and governments to buy carbon offsets to compensate for carbon dioxide emissions, while others are voluntary (for example, allowing individuals to offset greenhouse gas emissions when flying). These programmes are verified by certifying agencies such as the nonprofit organizations Verra and Gold Standard.

The Clean Development Mechanism is a potential platform for recognizing breastfeeding as a carbon offset. Implemented in 2005 when the 1997 Kyoto Protocol came into effect, the mechanism is the most important funding source for income redistribution between countries to address climate change. The mechanism is the main source of finance for the Adaptation Fund, which relies on contributions from the Clean Development Mechanism to support climate change adaptation projects in low- and middle-income countries that are parties to the Kyoto Protocol. Clean Development Mechanism projects must demonstrate greenhouse gas emission reductions while contributing to sustainable development as defined by the host country.^50^ In 2023, at the Conference of Parties 28 UN Climate Change Conference, parties agreed on the terms of the loss and damage mechanism.^51^ This mechanism has additional potential to support breastfeeding as a climate mitigation and adaptation measure. Several interventions exist that reliably increase breastfeeding rates at scale.^5^ The Green Feeding Tool is designed to meet accepted Clean Development Mechanism methods for measuring carbon offsets, and can estimate the carbon offset from public investments in policies or programmes that increase breastfeeding.^34^

Although one third of greenhouse gas emissions are produced by the global food system, the mechanism presently focuses most of its attention on energy use. In addition, its focus is on the supply side, that is, increasing the per unit energy efficiency of food production. This focus, however, fails to address total production and demand for unnecessary and unhealthy ultra-processed foods, which is increasing.^52^ As an example, focusing on reducing the energy used to produce a kilogram of commercial milk formula fails to consider the public and planetary health impacts of rising overall consumption. This productive efficiency focus has the concerning potential to help generate higher consumer demand through greenwashing, that is, company marketing communication that misleads consumers about environmental performance to promote product sales.^44^

Since producers and exporters of commercial milk formula are mostly based in high greenhouse gas-emitting countries, such a fund is a promising financial resource for low- and middle-income countries to adopt effective and human rights-based interventions that redress and repair the damage to breastfeeding practices done over many decades by commercial milk formula industry marketing and exports.^13^

We propose redirecting these resources to fund interventions that enable women and children to breastfeed. Reorienting financing through the Clean Development Mechanism and the global loss and damage fund in this way would simultaneously improve health and development and compensate for damage to the environment. Both women and children would benefit, as well as the global community. Adding breastfeeding investments to eligibility for climate financing would be a practical acknowledgement of women’s economic contributions, and would justly orient benefits towards governments and populations disproportionately burdened by the commercial milk formula industry’s social and ecological harms.

We can make a strong case to deploy the Clean Development Mechanism for commercial milk formula producer countries to provide financing for countries to invest in supporting high breastfeeding rates for the carbon offset achieved.^34^ A suitable international agency would audit and certify delivery of sufficiently effective programmes to pregnant and lactating women. Such initiatives would entail, for example, skilled birth attendance and adequate maternity care, and ending commercial milk formula marketing misinformation through full implementation of the International Code of Marketing of Breastmilk Substitutes. Efforts would also include social protections such as paid maternity leave entitlements, and breastfeeding-friendly work and childcare environments, along with suitable investments in community and household infrastructure so women have time for breastfeeding, good nutrition and self-care.^5^^,^^13^ Offset financing for investments enabling breastfeeding would be built around global and national ceilings on commercial milk formula sales per child (0–36 months), to reinforce policy priorities towards investments in breastfeeding and reduced greenhouse gas emissions. Ceilings would target diminishing commercial milk formula sales per child, aligned with achieving global breastfeeding targets. Monitoring of these indicators is already in place, but new global and national policy-making processes and governance arrangements that are free from commercial influence and conflicts of interest are critical to move along this pathway. To achieve this goal, governance arrangements must exclude, for example, companies that violate the International Code of Marketing of Breastmilk Substitutes.^13^

A call to consider breastfeeding as a carbon offset is not targeting women who choose not to breastfeed or who need to use commercial milk formula. Nor is it about coercing breastfeeding or shifting responsibility for climate change mitigation to those who are already overburdened, including by poverty. Rather, directing funding to governments that recognize the adverse greenhouse gas impacts of expanding commercial milk formula markets represents a gender-just transition to sustainable development, because individual women wanting to breastfeed will experience a more enabling environment. The carbon offset approach is intended to initiate a paradigm shift to reducing demand for unnecessary and unhealthy food products with high greenhouse gas emissions. This shift applies especially to heavily marketed follow-up formulas and growing-up milks promoted for ages 6 months and older, products that WHO considers entirely unnecessary for healthy infant and young child diets, yet account for at least half of global commercial milk formula sales.^53^

## Valuing breastfeeding 

Rethinking what matters is crucial to address interlocking crises and harmful influences on both human and planetary health.^18^ The report *Health for all: transforming economies to deliver what matters*^54^ provides much-needed impetus to radically reorient economic and financial policies so governments value breastfeeding and mothers’ milk for its nutrition, health and environmental benefits, and commercial determinants of ill-health are replaced. Governments’ initial responses to the COVID-19 pandemic illustrated that a full-spectrum holistic approach to finance, investment and governance is feasible and better able to value the health of people and planet across dimensions other than GDP.

The commercial milk formula industry’s expansion during the past several decades is based on a paradigm that prioritizes expansion of trade, commerce, financing and GDP growth in ways that reinforce the power of commercial determinants of health.

We propose a reorientation of systems of measurement and financing towards a new paradigm and metrics that would support health for all. In this view, women’s breastfeeding efforts would be valued for the multiple contributions to both human and planetary health.

Recognizing the value of breastfeeding as a carbon offset in redistributive funding initiatives like the Clean Development Mechanism illustrates a broad pathway towards human and planetary health, and sustainable development. Multiple co-benefits would arise from directing Clean Development Mechanism investments towards increasing breastfeeding.

Properly valuing crucial care and environmental resources in our economic measurement systems would redirect international financial resources away from expanding potentially harmful economic activity, and towards what really matters, including health for all. Doing so starts with breastfeeding as the local, sustainable and healthy first-food system for generations to come.
